# Assessing causality between obstructive sleep apnea with the dyslipidemia and osteoporosis: a Mendelian randomization study

**DOI:** 10.3389/fgene.2024.1359108

**Published:** 2024-06-20

**Authors:** Ping-Yang Hong, Dong Liu, Ang Liu, Xin Su, Xiao-Bin Zhang, Yi-Ming Zeng

**Affiliations:** ^1^ Department of Pulmonary and Critical Care Medicine, The Second Affiliated Hospital of Fujian Medical University, Center of Respiratory Medicine of Fujian, Quanzhou, China; ^2^ Department of Pulmonary and Critical Care Medicine, Zhongshan Hospital of Xiamen University, School of Medicine, Xiamen University, Xiamen, China; ^3^ Department of Cardiology, Xiamen Cardiovascular Hospital of Xiamen University, School of Medicine, Xiamen University, Xiamen, China; ^4^ Department of Civil Engineering and Smart Cities, Shantou University, Shantou, China; ^5^ Department of Anesthesiology, Heze Municipal Hospital, Heze, China; ^6^ The School of Clinical Medicine, Fujian Medical University, Fuzhou, China

**Keywords:** obstructive sleep apnea, dyslipidemia, mendelian randomization analysis, osteoporosis, assessing causality

## Abstract

**Purpose:**

This study aims to assess the causal relationship between Obstructive Sleep Apnea (OSA), dyslipidemia, and osteoporosis using Mendelian Randomization (MR) techniques.

**Methods:**

Utilizing a two-sample MR approach, the study examines the causal relationship between dyslipidemia and osteoporosis. Multivariable MR analyses were used to test the independence of the causal association of dyslipidemia with OSA. Single nucleotide polymorphisms (SNPs) were selected as instrumental variables based on genome-wide significance, independence, and linkage disequilibrium criteria. The data were sourced from publicly available Genome-Wide Association Studies (GWAS) of OSA (*n* = 375,657) from the FinnGen Consortium, the Global Lipids Genetics Consortium of dyslipidemia (*n* = 188,577) and the UK Biobank for osteoporosis (*n* = 456,348).

**Results:**

The MR analysis identified a significant positive association between genetically predicted OSA and triglyceride levels (OR: 1.15, 95% CI: 1.04–1.26, *p* = 0.006) and a negative correlation with high-density lipoprotein cholesterol (HDL-C) (OR: 0.84, 95% CI: 0.77–0.93, *p* = 0.0003). Conversely, no causal relationship was found between dyslipidemia (total cholesterol, triglycerides, HDL-C, and low-density lipoprotein cholesterol) and OSA or the relationship between OSA and osteoporosis.

**Conclusion:**

The study provides evidence of a causal relationship between OSA and dyslipidemia, highlighting the need for targeted prevention and management strategies for OSA to address lipid abnormalities. The absence of a causal link with osteoporosis and in the reverse direction emphasizes the need for further research in this area.

## Introduction

According to conservative estimates, the prevalence of obstructive sleep apnea (OSA) is 3% for women, 10% for men in the 30- to 49-year-old age range, and 9% for women and 17% for men in the 50- to 70-year-old age range (Peppard et al., 2013). The characteristic features of OSA include episodic collapse of the upper airway dependent on the sleep state (Gileles-Hillel et al., 2016). This leads to periodic decreases or cessations in breathing, which can cause hypoxia, hypercapnia, or arousal from sleep (Dempsey et al., 2010; Veasey and Rosen, 2019). These derangements result in changes related to the heart and metabolism, including dyslipidemia, osteoporosis, insulin resistance, hypertension, and atherosclerosis, which eventually raise the risk of cardiovascular morbidity and death (Barros and García-Río, 2019; Mesarwi et al., 2019; Gleeson and McNicholas, 2022). Dyslipidaemia is an independent risk factor for cardiovascular morbidity (Mach et al., 2020). There exists some evidence that links OSA to altered lipid profiles: patients with OSA frequently exhibit elevated concentrations of triglycerides (TG), total cholesterol (TC), and low-density lipoprotein cholesterol (LDL-C), along with a corresponding decrease in high-density lipoprotein cholesterol (HDL-C) levels (Li et al., 2007; Gündüz et al., 2018). Recent studies suggest a heightened risk of osteoporosis in individuals diagnosed with OSA (Chen et al., 2014; Daniel et al., 2022). OSA leads to chronic intermittent hypoxia, which can disrupt bone remodeling by affecting the balance between bone formation and resorption (Ananth and Schisterman, 2018). This imbalance can result in decreased bone mineral density. Additionally, the systemic inflammation associated with OSA may contribute to bone loss by promoting the activity of osteoclasts, the cells responsible for bone resorption (Sekula et al., 2016).

However, the causal relationship between OSA, dyslipidemia, and osteoporosis remains unknown. It is challenging to determine the causative relationship between OSA, dyslipidemia, and osteoporosis for the correlation seen in observational research because of the possible confounding biases and reverse causation present in these investigations (Ananth and Schisterman, 2018).

Utilizing genetic variants as instrumental variables for risk factors, Mendelian randomization (MR) design assesses the causal relationship between risk factors and disease (Sekula et al., 2016). MR analysis can eliminate potential unmeasured confounders and reverse causation, a significant limitation of evidence from observational studies because the genetic variants are assigned randomly at conception (Bowden and Holmes, 2019). In this work, we used MR techniques to assess the causal relationship between OSA, dyslipidemia, and osteoporosis.

## Methods

### Study design and data sources

**FIGURE 1:**
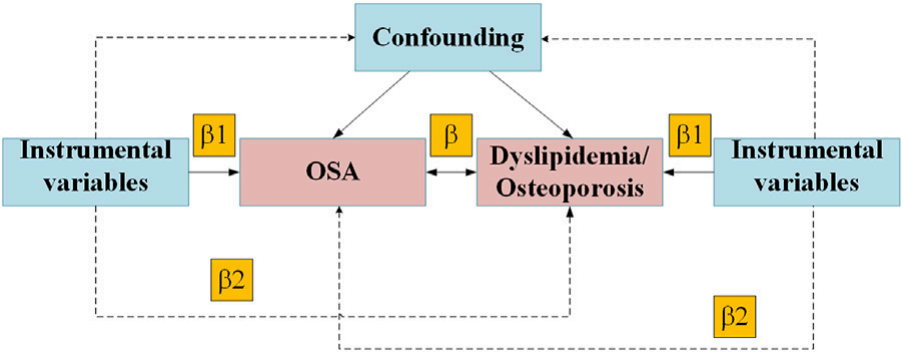
Mendelian randomization model of OSA, Dyslipidemia and Osteoporosis. Abbreviation: OSA, obstructive sleep apnea.

This study encompasses a comprehensive review of Supplementary Material within the article. We employed a two-sample MR approach to investigate the causal relationship between OSA, dyslipidemia, and osteoporosis (Figure 1). In our MR framework, genetic variations serve as instrumental variables to ascertain if exposure significantly influences disease development. This method offers robust causal inferences, mitigating the impact of unmeasured confounders. Our MR design adhered to three critical criteria for credible causal estimations: 1. Instrumental variables must exhibit a substantial association with the exposure; 2. The instrumental variables should be independent of known confounders. The exposure is the sole pathway through which the instrumental variables influence the outcomes; 3. Genome-wide association studies (GWAS) have demonstrated associations between Single nucleotide polymorphisms (SNPs) and dyslipidemia. Only summary data were used in this article. Appropriate ethical approval and patient informed consent were obtained in the original studies. GWAS data for OSA were obtained from the FinnGen Consortium (G6_SLEEPAPNO), comprising 38,998 cases and 336,659 controls. Genetic instruments for lipid traits of 188,577 participants included serum triglycerides (TG), serum total cholesterol (TC), serum low-density lipoprotein cholesterol (LDL-C), and serum high-density lipoprotein cholesterol (HDL-C) were sourced from the Global Lipids Genetics Consortium (GLGC) (available at [http://www.lipidgenetics.org/]) (Willer et al., 2013). GWAS data for osteoporosis were obtained from the UK Biobank (n = 456,348) (Table 1).

**TABLE 1:** Details of the GWASs included in the Mendelian randomization.

Consortium	Phenotype	Participants			Web source
		Ncase		ncontrl	
FinnGen	OSA	38,998		336,659	https://r9.finngen.fi/
Global Lipids Genetics Consortium	Level of lipids	188,577			https://csg.sph.umich.edu/willer/public/lipids2013/
UK Biobank	Osteoporosis	991		455,357	https://www.ebi.ac.uk/gwas/studies/GCST90044600

Abbreviation: OSA, obstructive sleep apnea.

### Instrumental variable selection

OSA was diagnosed according to International Classification of Diseases, 10th Revision (ICD-10) and Ninth Revision (ICD-9) codes (ICD-10:G47.3, ICD-9:3472A), which is based on subjective symptoms, clinical examination and sleep registration applying apnea-hypopnea index ≥5/hour or respiratory event index ≥5/hour. In this study, SNPs were meticulously selected for each exposure factor in accordance with the principal assumptions underpinning MR. Initially, SNPs achieving genome-wide significance (*p* < 5 × 10^−8) were considered for inclusion. Subsequently, to identify independent instrumental variables (IVs), we selected variants demonstrating the lowest *p*-values, ensuring minimal linkage disequilibrium (LD) as evidenced by an *r*^2 threshold greater than 0.1, based on the European 1000 Genome reference panel. Finally, the robustness of these instrumental variables was quantified using *F*-statistics (Burgess et al., 2011), with an *F*-statistic value exceeding ten generally deemed suitable for MR analysis.

### Statistical analysis

In this investigation, for binary exposures, causal estimates were articulated as odds ratios (ORs) with 95% confidence intervals (CIs) per logarithmic odds increment in the genetically predisposed risk of the exposures. Regarding continuous exposures, the causal estimate was denoted as an OR accompanied by a 95% CI for each standard deviation (SD) increase in exposure. MR analysis employed the primary analytic approach of the random-effects inverse-variance weighted (IVW) method. This was chosen to estimate the potential bidirectional causal relationships between OSA and dyslipidemia, offering robust causal estimations in scenarios devoid of directional pleiotropy. Complementary analyses incorporated methods such as the weighted median, simple mode, weighted mode, and MR-Egger. Directional horizontal pleiotropy was assessed using the MR-Egger intercept test. Heterogeneity in MR-Egger regression and the IVW method was evaluated through Cochran’s Q statistics and funnel plot analyses. (Bowden et al., 2018). Additionally, sensitivity was examined via leave-one-out analysis. Post hoc power assessments for MR leveraged online resources (https://sb452.shinyapps.io/power/) (Burgess, 2014). All statistical procedures were executed using the TwoSampleMR packages within R (version 4.1.2, www.r-project.org/). All *p*-values were two-tailed. A Bonferroni-adjusted *p*-value threshold of <0.004 (0.05/12) was set for determining statistical significance in MR analyses. In contrast, *p*-values <0.10 were deemed significant for MR-Egger tests and heterogeneity assessments.

## Results

### Instrumental variable selection

In the initial phase of our analysis, we rigorously selected SNPs that demonstrated a robust association with the exposure, applying stringent criteria (*p* < 5 × 10^−8, F-value >10) and ensuring independence (*r* ^2^ < 0.001 within a 10,000 kb physical window). This process yielded 24 SNPs from the FinnGen Consortium (G6_SLEEPAPNO) and respective 88, 70, 101, and 82 SNPs from the GLGC for TC, TG, HDL-C, and LDL-C, preliminarily designated as IVs. Further refinement was conducted using Phenoscanner V2 to exclude SNPs associated with outcomes or confounders (*p* < 1 × 10^−5). This led to removing 2 SNPs from the FinnGen Consortium (G6_SLEEPAPNO) 5, 4, 7, and 5 SNPs from the GLGC for TC, TG, HDL-C, and LDL-C, respectively. These exclusions were pivotal in preparing the remaining SNPs for subsequent inclusion in the Mendelian randomization analysis.

### The causal effect of OSA on dyslipidemia

**FIGURE 2:**
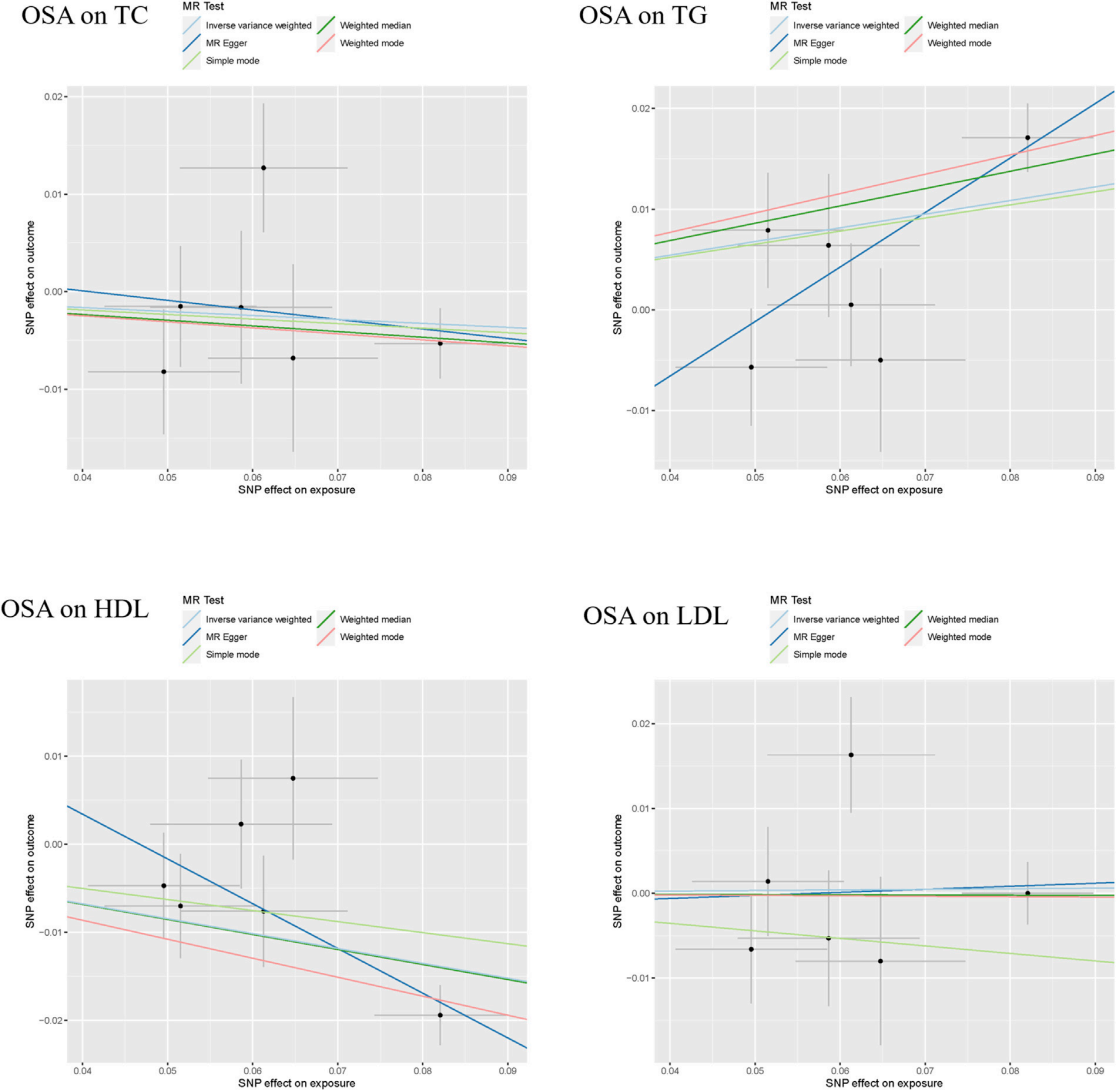
The scatter plots of the association between genetically predicted OSA and dyslipidemia. Abbreviation: TG, serum triglycerides; TC, serum total cholesterol; LDL-C, serum low-density lipoprotein cholesterol; HDL-C, serum high-density lipoprotein cholesterol.

The results of the MR analyses are shown in Table 2, and the scatter plots and forest plots are presented in Figure 2 and Supplementary Figure S1, respectively. Genetically predicted OSA was significantly positively associated with TG. The OR with 95% CI of per log-odds increment in OSA liability was 1.15 (95% CI: 1.04–1.26; *p* = 0.006) in the IVW model, which was consistent with the result of the MR Egger model, weighted median model, and weighted model. The Cochran’s Q value suggested a moderate level of heterogeneity (Q = 11.677, *p* < 0.05) obtained from individual variants. Furthermore, the leave-one-out analysis suggested that the observed association was not significantly changed after removing any single variant (Supplementary Figure S2). Regarding HDL-C, a negative correlation was noted with genetic predisposition to OSA. The ORs per log-odds increase in genetically inferred OSA were 0.84 (95% CI: 0.77–0.93; *p* = 0.0003), consistent across the MR Egger, weighted median, and weighted models. Cochran’s Q value suggested an absence of heterogeneity (*p* > 0.05) across the variants. Leave-one-out analysis further supported the stability of this association, as detailed in Supplementary Figure S2.

**TABLE 2:** MR results for the relationship between OSA on level of lipids.

Exposures	Outcomes	No. of SNPs	Method	OR (95%CI)	*p*	Heterogeneity test		Pleiotropy test
						Cochran’s Q (*I* ^2^)	*p*	*p* pintercept
OSA*	TC^#^	22	IVW MR Egger Weighted median Simple mode Weighted mode	0.96 (0.89–1.04) 0.91 (0.59–1.41) 0.94 (0.87–1.02) 0.95 (0.83–1.09) 0.94 (0.86–1.03)	0.312 0.685 0.146 0.516 0.229	6.738 (25.79%) 6.625 (39.62%)	0.241 0.157	0.807
OSA*	TG^#^	22	IVW	1.15 (1.04–1.26)	0.006	11.677 (57.18%)	0.039	0.085
			MR Egger	1.71 (1.20–2.46)	0.041	5.095 (21.50%)	0.278	
			Weighted median	1.19 (1.09–1.29)	5.81e-05			
			Simple mode	1.14 (0.91–1.42)	0.305			
			Weighted mode	1.21 (1.11–1.32)	0.006			
OSA*	HDL^#^	22	IVW	0.84 (0.77–0.93)	0.0003	10.114 (50.56%)	0.072	0.151
			MR Egger	0.60 (0.41–0.88)	0.060	5.665 (29.39%)	0.226	
			Weighted median	0.84 (0.78–0.92)	6.52e-05			
			Simple mode	0.88 (0.72–1.08)	0.271			
			Weighted mode	0.81 (0.74–0.88)	0.005			
OSA*	LDL^#^	22	IVW	1.01 (0.92–1.10)	0.883	7.915 (36.83%)	0.161	0.910
			MR Egger	1.04 (0.64–1.69)	0.892	7.886 (49.28%)	0.096	
			Weighted median	1.00 (0.92–1.08)	0.946			
			Simple mode	0.92 (0.78–1.07)	0.330			
			Weighted mode	0.99 (0.92–1.08)	0.906			
OSA*	Osteoporosis^&^	21	IVW	0.83 (0.52–1.35)	0.461	23.459 (14.75%)	0.218	0.978
			MR Egger	0.81 (0.10–6.78)	0.848	23.460 (19.01%)	0.267	
			Weighted median	0.74 (0.38–1.44)	0.380			
			Simple mode	0.68 (0.19–2.44)	0.561			
			Weighted mode	0.46 (0.15–1.38)	0.184			

^*^Data form The FinnGen Consortium (G6_SLEEPAPNO).

^#^Data form Global Lipids Genetics Consortium.

^&^Data form UK, biobank.

Abbreviation: OSA, obstructive sleep apnea; SNPs, single-nucleotide polymorphisms; TG, triglycerides; TC, total cholesterol; LDL-C, low-density lipoprotein cholesterol; HDL-C, high-density lipoprotein cholesterol; IVW, inverse-variance weighted.

### The causal effect of dyslipidemia on OSA

As shown in Table 3, the scatter plots (Supplementary Figure S4), and forest plots (Supplementary Figure S5), the MR results showed TC, TG, HDL-C, and LDL-C were not causally related to OSA, with ORs close to 1 (*p* > 0.05). Egger’s test showed that no potential horizontal pleiotropy exists except for the relationship between TG and the risk of OSA. Cochran’s Q test indicated obvious heterogeneities. The leave-one-out analysis also revealed the stability of the results (Supplementary Figure S6).

**TABLE 3:** MR results for the relationship between dyslipidemia and Gout on OSA.

Exposures	Outcomes	No. of SNPs	Method	OR (95%CI)	*p*	Heterogeneity test		Pleiotropy test
						Cochran’s Q (*I* ^2^)	*p*	*p* pintercept
TC^#^	OSA*	83	IVW MR Egger Weighted median Simple mode Weighted mode	0.99 (0.95–1.04) 1.00 (0.92–1.08) 0.99 (0.94–1.06) 1.01 (0.91–1.13) 0.99 (0.94–1.05)	0.750 0.941 0.842 0.798 0.798	139.793 (41.34%) 139.761 (42.04%)	5.49e-05 7.29e-05	0.892
TG^#^	OSA*	66	IVW	1.04 (0.95–1.14)	0.396	225.735 (71.21%)	9.40e-18	0.046
			MR Egger	0.92 (0.80–1.07)	0.283	212.059 (69.82%)	1.30e-19	
			Weighted median	0.99 (0.92–1.06)	0.754			
			Simple mode	1.02 (0.89–1.16)	0.821			
			Weighted mode	0.99 (0.93–1.06)	0.810			
HDL^#^	OSA*	94	IVW	0.96 (0.91–1.01)	0.122	167.779 (44.57%)	3.28e-06	0.709
			MR Egger	0.97 (0.88–1.08)	0.601	167.523 (45.08%)	2.54e-06	
			Weighted median	0.93 (0.87–0.99)	0.299			
			Simple mode	0.99 (0.87–1.13)	0.857			
			Weighted mode	0.94 (0.88–1.00)	0.608			
LDL^#^	OSA*	77	IVW	1.00 (0.97–1.04)	0.871	100.972 (24.73%)	0.029	0.974
			MR Egger	1.00 (0.95–1.06)	0.892	100.971 (25.72%)	0.024	
			Weighted median	1.00 (0.95–1.05)	0.917			
			Simple mode	1.03 (0.94–1.13)	0.555			
			Weighted mode	1.00 (0.96–1.05)	0.896			

^#^Data form Global Lipids Genetics Consortium

^*^Data form The FinnGen Consortium (G6_SLEEPAPNO)

Abbreviation: OSA, obstructive sleep apnea; SNPs, single-nucleotide polymorphisms; TG, triglycerides; TC, total cholesterol; LDL-C, low-density lipoprotein cholesterol; HDL-C, high-density lipoprotein cholesterol; IVW, inverse-variance weighted.

**FIGURE 3:**
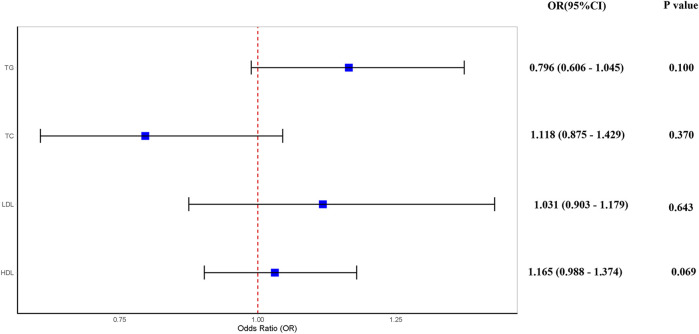
Multivariate Mendelian randomization analysis of the causal effect of dyslipidemia on OSA. Abbreviation: TG, serum triglycerides; TC, serum total cholesterol; LDL-C, serum low-density lipoprotein cholesterol; HDL-C, serum high-density lipoprotein cholesterol.

Considering the inherent interconnections among TC, TG, HDL-C, and LDL-C, we conducted a comprehensive multivariate MR to elucidate the association between lipid profiles and OSA. Our findings indicate that there is no causal link between TC, TG, HDL-C, and LDL-C levels and the development of OSA (*p* > 0.05) (Figure 3).

### The causal effect of OSA on osteoporosis

The analysis revealed no causal link between OSA and osteoporosis, as evidenced by *p* values exceeding 0.05 (Table 2). The result in the IVW model was consistent with the result of the MR Egger model, weighted median model, and weighted model. Additionally, the limited number of SNPs precluded the feasibility of conducting a reverse analysis.

## Discussion

In this bidirectional two-sample MR study, we found that genetically predicted OSA was significantly positively associated with TG while negatively associated with HDL-C. Conversely, the reverse MR analyses yielded no substantial evidence suggesting any association of liability to TC, TG, HDL-C, and LDL-C with OSA. The findings indicate a lack of causal connection between OSA and osteoporosis.

Epidemiologic data consistently suggest that OSA positively correlates with dyslipidemia and osteoporosis (Phillips et al., 2011; Chen et al., 2014; Nadeem et al., 2014; Mesarwi et al., 2019). Several meta-analyses of cohort studies have indicated OSA as an independent risk factor for this condition, aligning with our results (Xu et al., 2014; Gündüz et al., 2018). However, observational studies examining the impact of dyslipidemia on OSA risk remain inconclusive. Our Mendelian Randomization (MR) analysis found no substantial evidence to support a definitive causal effect of dyslipidemia on OSA.

While the precise mechanisms linking OSA and dyslipidemia are not fully elucidated, several hypotheses have been proposed. Firstly, individuals suffering from OSA tend to have lifestyles characterized by minimal physical activity and diets that are heavy in fats and carbohydrates, which might escalate the risk of lipid metabolism dysfunction. (Jonassen et al., 2022). Second, studies from rodent models indicate that the hypoxic burden associated with OSA may play a major role in developing this impairment. Chronic intermittent hypoxia in obese mice, for example, has been shown to raise the content of liver triglycerides, upregulate hepatic biosynthetic pathways, and increase total cholesterol and low-density lipoprotein in lean mice (Li et al., 2005; Jun et al., 2010). The study did not establish a causal link between OSA and osteoporosis. While the MR study conducted did not find a genetic causal relationship between OSA and osteoporosis, this finding does not negate the possible clinical and biological connections observed in epidemiological studies. It is important to consider the limitations of MR studies, such as the possibility of pleiotropic effects of genetic variants or insufficient power to detect a true effect if the genetic instruments do not fully capture the pathological pathways of OSA impacting bone health. More detailed mechanistic studies are needed to explore how intermittent hypoxia and other physiological changes associated with OSA specifically affect bone metabolism. Further longitudinal studies with larger cohorts and better controls for confounding factors are required to clarify the relationship between OSA and changes in bone density over time. Clinical trials investigating the impact of effective OSA treatment (such as CPAP therapy) on bone health outcomes could provide insights into whether mitigating OSA could also benefit bone density and reduce the risk of osteoporosis. Our findings demonstrate a significant causal association between OSA and alterations in lipid profiles, specifically elevated TC levels and reduced HDL-C. These results support the recommendation for routine lipid screening in patients diagnosed with OSA. Appropriate interventions, including lifestyle modifications and pharmacological treatments targeting dyslipidemia, could be beneficial. Such measures are likely to mitigate the enhanced cardiovascular risk attributed to dyslipidemic states in individuals with OSA. Although our study did not establish a causal link between OSA and osteoporosis, it highlights the necessity for additional research in this domain. Investigating this potential association remains clinically significant as identifying a causal relationship could inform more integrated care approaches that concurrently address both respiratory and skeletal health in patients with OSA.

A major strength of this MR study lies in its ability to circumvent reverse causality and minimize residual confounding. Additionally, the study boasts significant investigatory power and accuracy in estimating effect magnitudes by employing the most comprehensive dataset for exposures and the most extensive summary-level data for OSA, dyslipidemia, and osteoporosis. Nonetheless, there are limitations. Firstly, the functions of the genetic instruments and their impact on risk factors are not completely understood. Secondly, potential pleiotropic effects, possibly obscured by a limited number of genetic instruments or small sample sizes, remain a concern, although the MR-Egger intercept indicates minimal horizontal pleiotropy.

In conclusion, our bidirectional MR study suggests a causal relationship between OSA and dyslipidemia, with no evidence of causality in the reverse direction or the causal relationship between OSA and osteoporosis. These findings underscore the importance of enhancing prevention, management, and treatment strategies for OSA to address dyslipidemia.

## Data Availability

The original contributions presented in the study are included in the article/Supplementary Material, further inquiries can be directed to the corresponding authors.

## References

[B1] Ananth C. V. Schisterman E. F. (2018). Hidden biases in observational epidemiology: the case of unmeasured confounding. Bjog 125 (6), 644–646. 10.1111/1471-0528.14960 29030998 PMC6166878

[B2] Barros D. García-Río F. (2019). Obstructive sleep apnea and dyslipidemia: from animal models to clinical evidence. Sleep 42 (3), zsy236. 10.1093/sleep/zsy236 30476296

[B3] Bowden J. Holmes M. V. (2019). Meta-analysis and Mendelian randomization: a review. Res. Synth. Methods 10 (4), 486–496. 10.1002/jrsm.1346 30861319 PMC6973275

[B4] Bowden J. Spiller W. Del Greco M. F. Sheehan N. Thompson J. Minelli C. (2018). Improving the visualization, interpretation and analysis of two-sample summary data Mendelian randomization via the Radial plot and Radial regression. Int. J. Epidemiol. 47 (4), 1264–1278. 10.1093/ije/dyy101 29961852 PMC6124632

[B5] Burgess S. (2014). Sample size and power calculations in Mendelian randomization with a single instrumental variable and a binary outcome. Int. J. Epidemiol. 43 (3), 922–929. 10.1093/ije/dyu005 24608958 PMC4052137

[B6] Burgess S. Thompson S. G. CRP CHD Genetics Collaboration (2011). Avoiding bias from weak instruments in Mendelian randomization studies. Int. J. Epidemiol. 40 (3), 755–764. 10.1093/ije/dyr036 21414999

[B7] Chen Y. L. Weng S. F. Shen Y. C. Chou C. W. Yang C. Y. Wang J. J. (2014). Obstructive sleep apnea and risk of osteoporosis: a population-based cohort study in Taiwan. J. Clin. Endocrinol. Metab. 99 (7), 2441–2447. 10.1210/jc.2014-1718 24735427

[B8] Daniel S. Cohen-Freud Y. Shelef I. Tarasiuk A. (2022). Bone mineral density alteration in obstructive sleep apnea by derived computed tomography screening. Sci. Rep. 12 (1), 6462. 10.1038/s41598-022-10313-w 35440678 PMC9018731

[B9] Dempsey J. A. Veasey S. C. Morgan B. J. O'Donnell C. P. (2010). Pathophysiology of sleep apnea. Physiol. Rev. 90 (1), 47–112. 10.1152/physrev.00043.2008 20086074 PMC3970937

[B10] Gileles-Hillel A. Kheirandish-Gozal L. Gozal D. (2016). Biological plausibility linking sleep apnoea and metabolic dysfunction. Nat. Rev. Endocrinol. 12 (5), 290–298. 10.1038/nrendo.2016.22 26939978

[B11] Gleeson M. McNicholas W. T. (2022). Bidirectional relationships of comorbidity with obstructive sleep apnoea. Eur. Respir. Rev. 31 (164), 210256. 10.1183/16000617.0256-2021 35508332 PMC9488957

[B12] Gündüz C. Basoglu O. K. Hedner J. Zou D. Bonsignore M. R. Hein H. (2018). Obstructive sleep apnoea independently predicts lipid levels: data from the European Sleep Apnea Database. Respirology 23 (12), 1180–1189. 10.1111/resp.13372 30133061

[B13] Jonassen T. M. Bjorvatn B. Saxvig I. W. Eagan T. M. Lehmann S. (2022). Clinical information predicting severe obstructive sleep apnea: a cross-sectional study of patients waiting for sleep diagnostics. Respir. Med. 197, 106860. 10.1016/j.rmed.2022.106860 35490509

[B14] Jun J. Reinke C. Bedja D. Berkowitz D. Bevans-Fonti S. Li J. (2010). Effect of intermittent hypoxia on atherosclerosis in apolipoprotein E-deficient mice. Atherosclerosis 209 (2), 381–386. 10.1016/j.atherosclerosis.2009.10.017 19897196 PMC2846209

[B15] Li J. Grigoryev D. N. Ye S. Q. Thorne L. Schwartz A. R. Smith P. L. (2005). Chronic intermittent hypoxia upregulates genes of lipid biosynthesis in obese mice. J. Appl. Physiol. (1985) 99 (5), 1643–1648. 10.1152/japplphysiol.00522.2005 16037401

[B16] Li J. Savransky V. Nanayakkara A. Smith P. L. O'Donnell C. P. Polotsky V. Y. (2007). Hyperlipidemia and lipid peroxidation are dependent on the severity of chronic intermittent hypoxia. J. Appl. Physiol. (1985) 102 (2), 557–563. 10.1152/japplphysiol.01081.2006 17082365

[B17] Mach F. Baigent C. Catapano A. L. Koskinas K. C. Casula M. Badimon L. (2020). 2019 ESC/EAS Guidelines for the management of dyslipidaemias: lipid modification to reduce cardiovascular risk. Eur. Heart J. 41 (1), 111–188. 10.1093/eurheartj/ehz455 31504418

[B18] Mesarwi O. A. Loomba R. Malhotra A. (2019). Obstructive sleep apnea, hypoxia, and nonalcoholic fatty liver disease. Am. J. Respir. Crit. Care Med. 199 (7), 830–841. 10.1164/rccm.201806-1109TR 30422676 PMC6835083

[B19] Nadeem R. Singh M. Nida M. Waheed I. Khan A. Ahmed S. (2014). Effect of obstructive sleep apnea hypopnea syndrome on lipid profile: a meta-regression analysis. J. Clin. Sleep. Med. 10 (5), 475–489. 10.5664/jcsm.3690 24910548 PMC4046360

[B20] Peppard P. E. Young T. Barnet J. H. Palta M. Hagen E. W. Hla K. M. (2013). Increased prevalence of sleep-disordered breathing in adults. Am. J. Epidemiol. 177 (9), 1006–1014. 10.1093/aje/kws342 23589584 PMC3639722

[B21] Phillips C. L. Yee B. J. Marshall N. S. Liu P. Y. Sullivan D. R. Grunstein R. R. (2011). Continuous positive airway pressure reduces postprandial lipidemia in obstructive sleep apnea: a randomized, placebo-controlled crossover trial. Am. J. Respir. Crit. Care Med. 184 (3), 355–361. 10.1164/rccm.201102-0316OC 21527567

[B22] Sekula P. Del Greco M. F. Pattaro C. Köttgen A. (2016). Mendelian randomization as an approach to assess causality using observational data. J. Am. Soc. Nephrol. 27 (11), 3253–3265. 10.1681/ASN.2016010098 27486138 PMC5084898

[B23] Veasey S. C. Rosen I. M. (2019). Obstructive sleep apnea in adults. N. Engl. J. Med. 380 (15), 1442–1449. 10.1056/NEJMcp1816152 30970189

[B24] Willer C. J. Schmidt E. M. Sengupta S. Peloso G. M. Gustafsson S. Kanoni S. (2013). Discovery and refinement of loci associated with lipid levels. Nat. Genet. 45 (11), 1274–1283. 10.1038/ng.2797 24097068 PMC3838666

[B25] Xu H. Yi H. Guan J. Yin S. (2014). Effect of continuous positive airway pressure on lipid profile in patients with obstructive sleep apnea syndrome: a meta-analysis of randomized controlled trials. Atherosclerosis 234 (2), 446–453. 10.1016/j.atherosclerosis.2014.03.034 24769714

