# Supplementary material for: Assessing causality between obstructive sleep apnea with the dyslipidemia and osteoporosis: a Mendelian randomization study
Source: Front Genet. 2024 Jun 20;15:1359108. doi: 10.3389/fgene.2024.1359108 (PMC11222592; doi:10.3389/fgene.2024.1359108)

Supplementary materials

Assessing Causality Between Obstructive Sleep Apnea with the Dyslipidemia and Osteoporosis: A Mendelian Randomization Study

Ping-Yang Hong, Ang Liu, Xin Su, Xiao-Bin Zhang, Yi-Ming Zeng

Contents

Figure S1. Forest plot of variant specific inverse variance estimates for causal association between OSA on Dyslipidemia. 2

Figure S2. Leave-one-out plot to assess if a single SNP is driving the association between OSA and Dyslipidemia. 3

Figure S3. Funnel plot of causal association between OSA on Dyslipidemia 4

Figure S4. The scatter plots of the association between genetically predicted Dyslipidemia on OSA 5

Figure S5. Forest plot of variant specific inverse variance estimates for causal association between Dyslipidemia on OSA. 6

Figure S6. Leave-one-out plot to assess if a single SNP is driving the association between Dyslipidemia and OSA. 7

Figure S7. Funnel plot of causal association between Dyslipidemia on OSA 8

Figure S8 MR Results for the Relationship Between OSA on Osteoporosis 9

Figure S1. Forest plot of variant specific inverse variance estimates for causal association between OSA on Dyslipidemia.

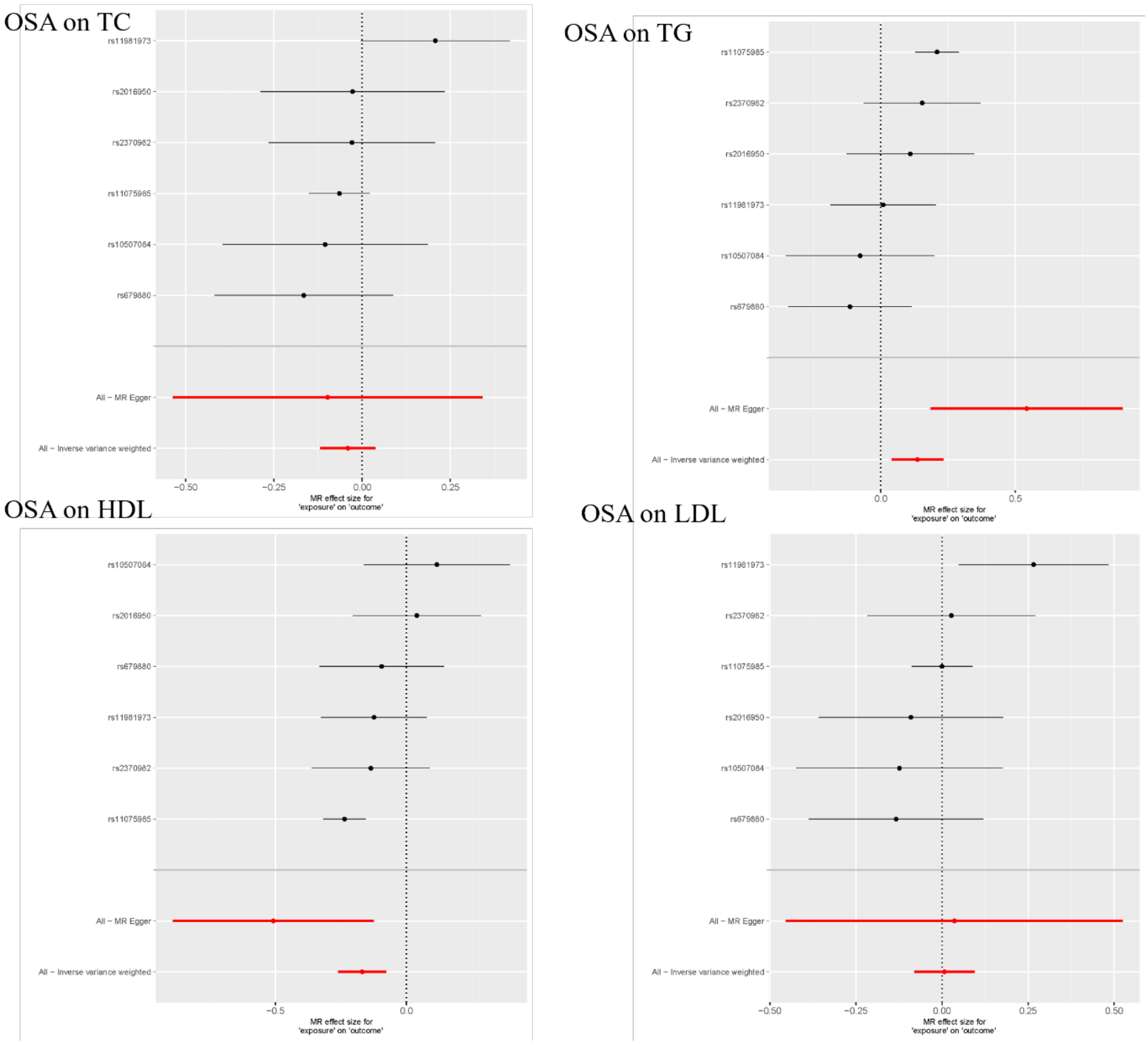

Figure S2. Leave-one-out plot to assess if a single SNP is driving the association between OSA and Dyslipidemia.

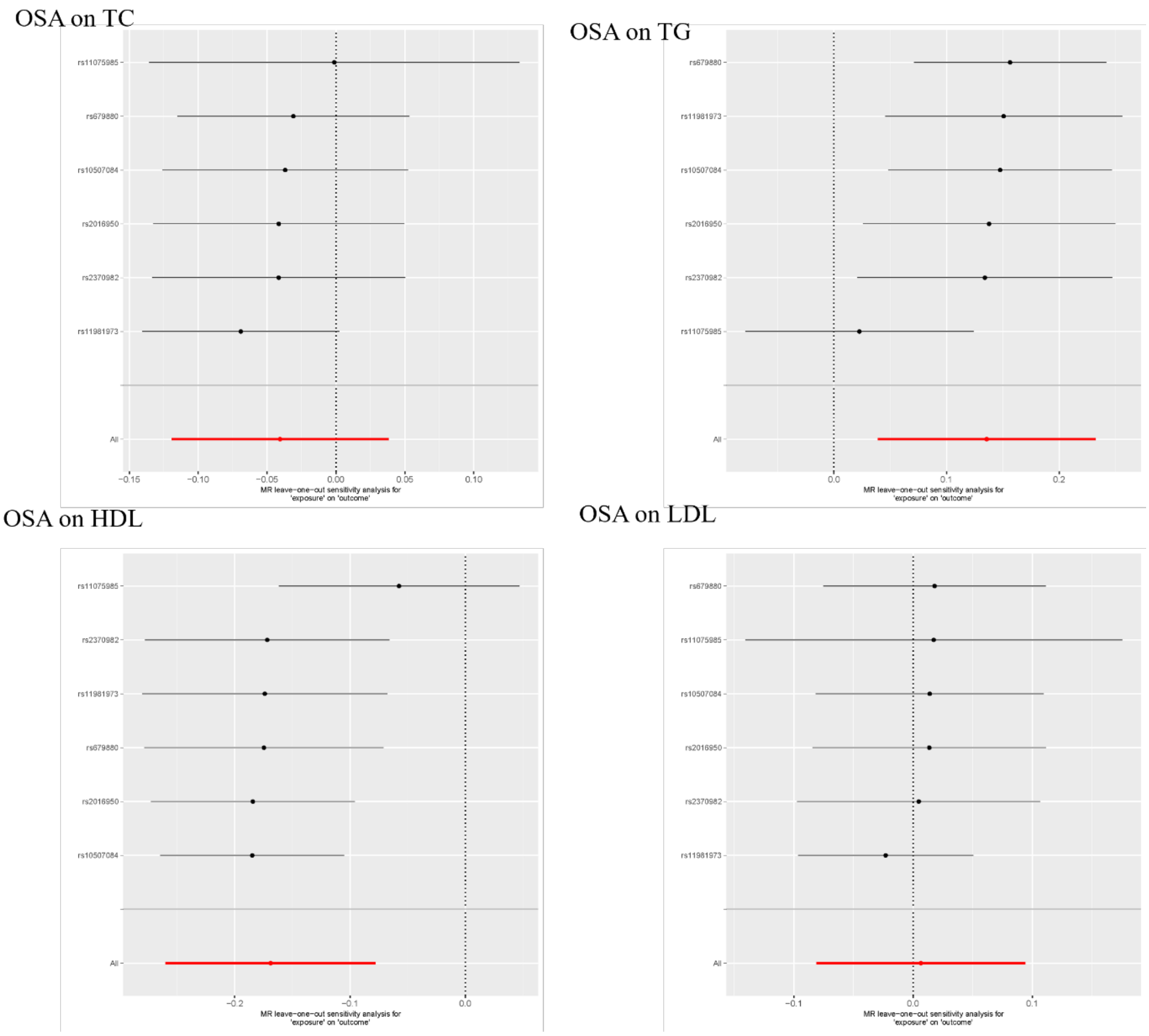

Figure S3. Funnel plot of causal association between OSA on Dyslipidemia

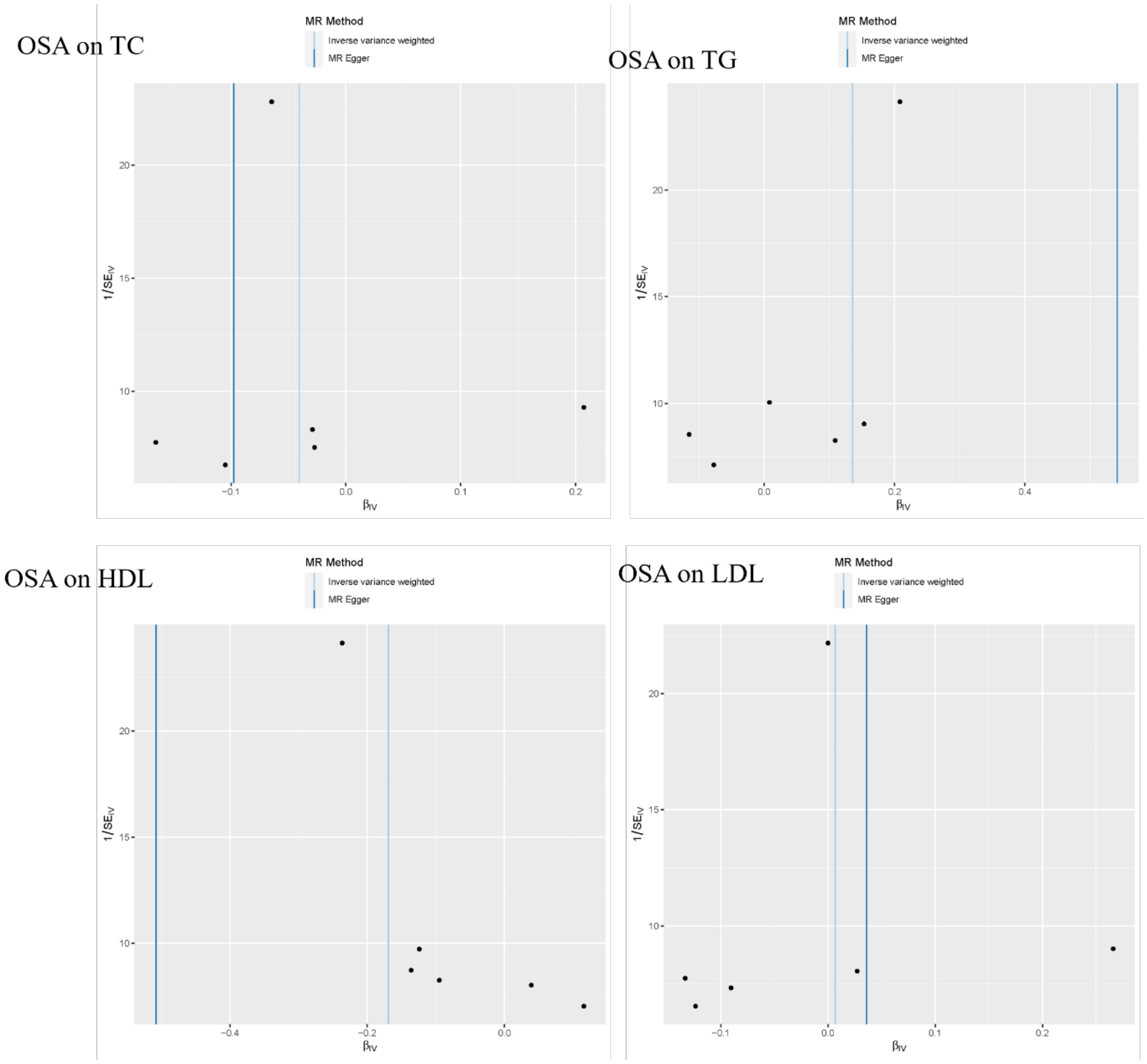

Figure S4. The scatter plots of the association between genetically predicted Dyslipidemia on OSA.

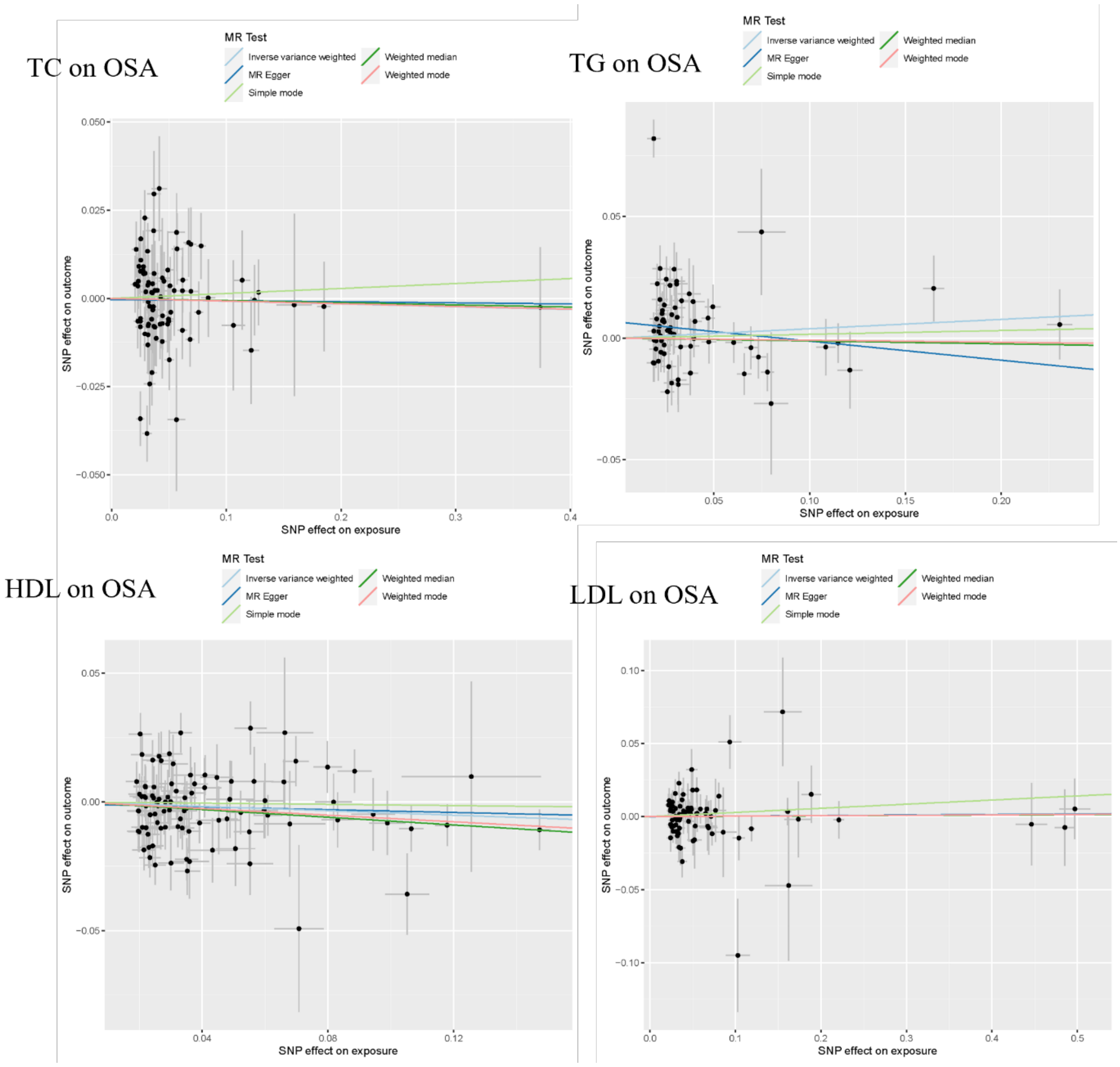

Figure S5. Forest plot of variant specific inverse variance estimates for causal association between Dyslipidemia on OSA.

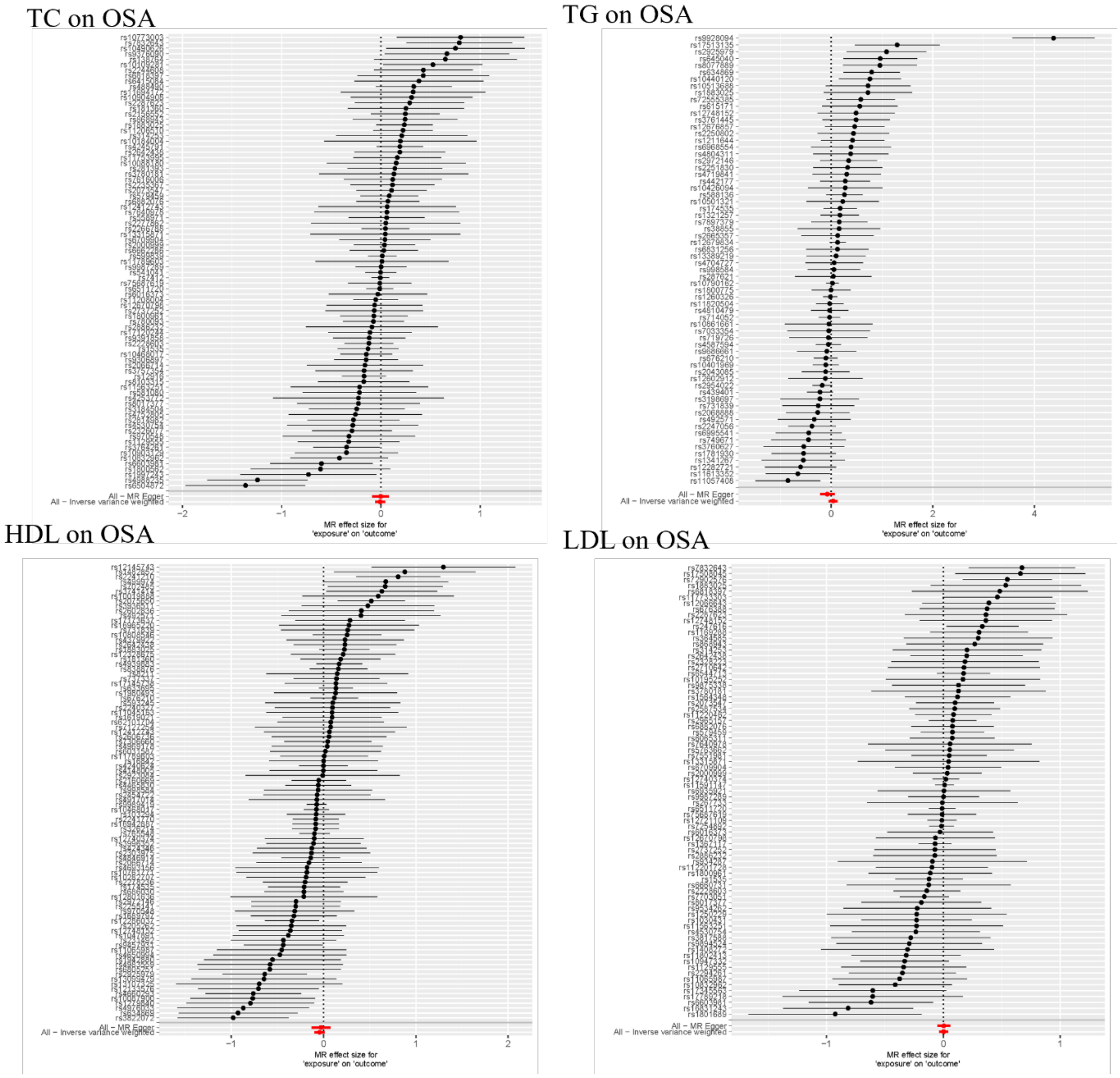

Figure S6. Leave-one-out plot to assess if a single SNP is driving the association between Dyslipidemia and OSA.

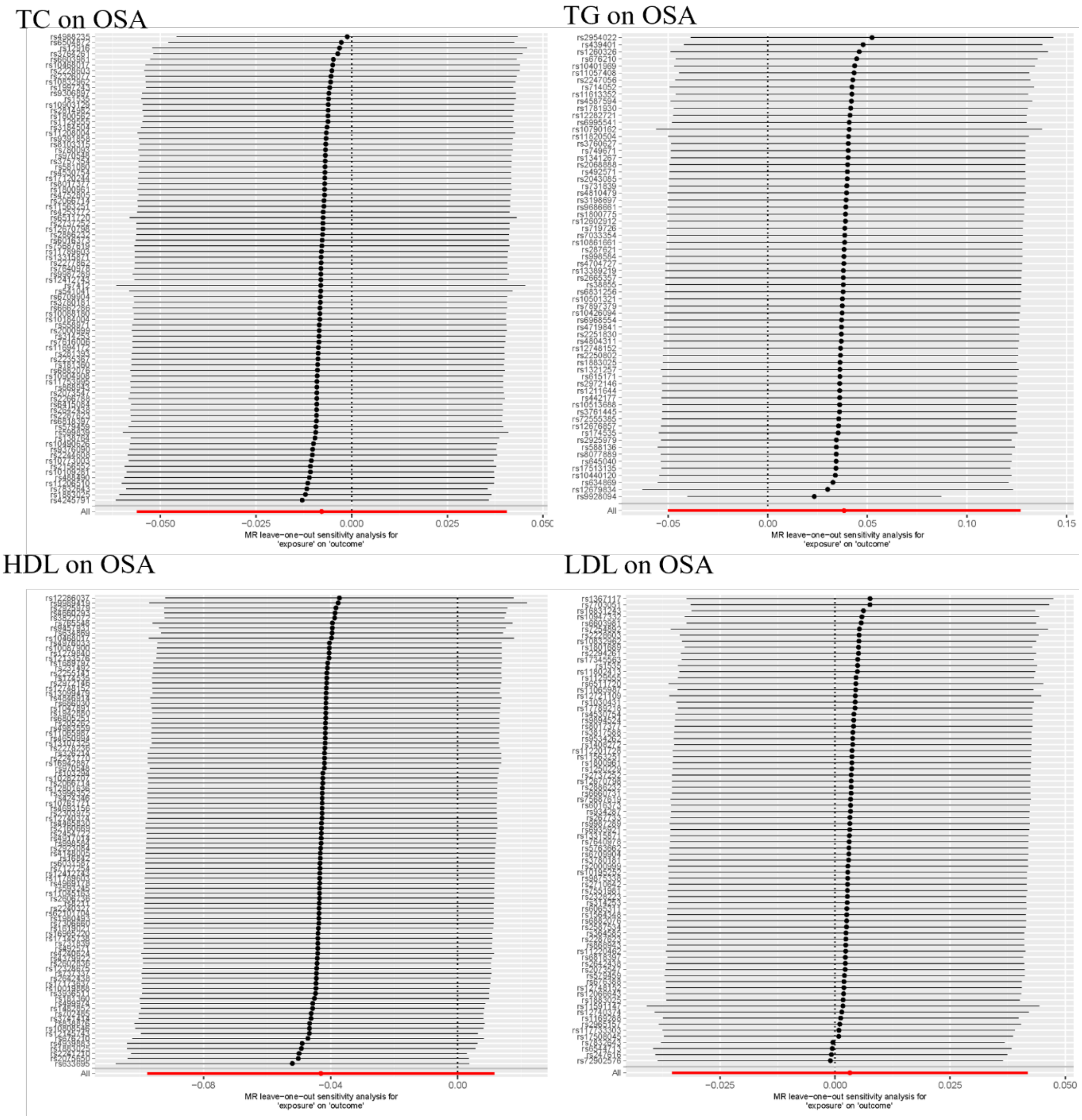

Figure S7. Funnel plot of causal association between Dyslipidemia on OSA.

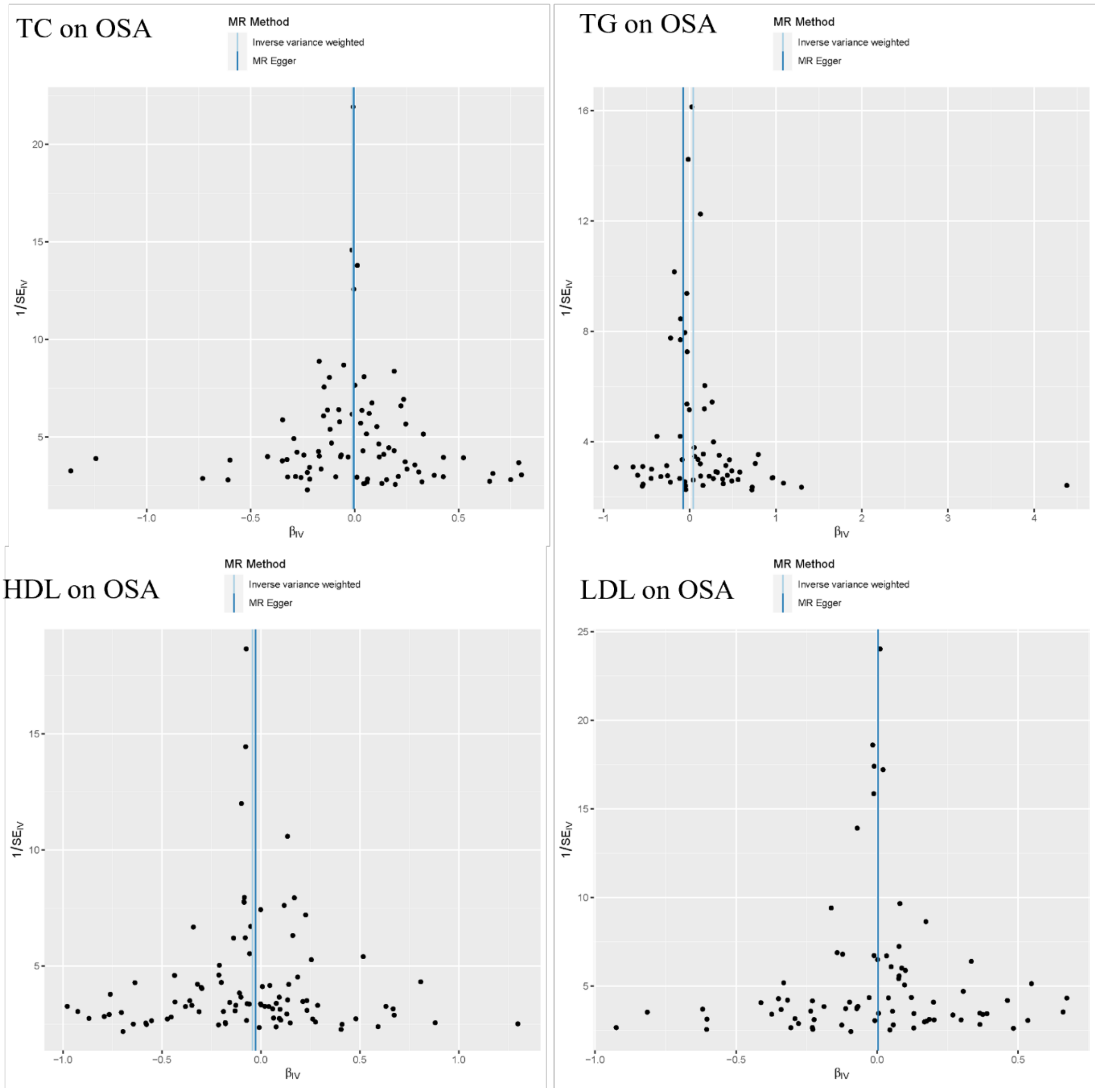

Figure S8 MR Results for the Relationship Between OSA on Osteoporosis

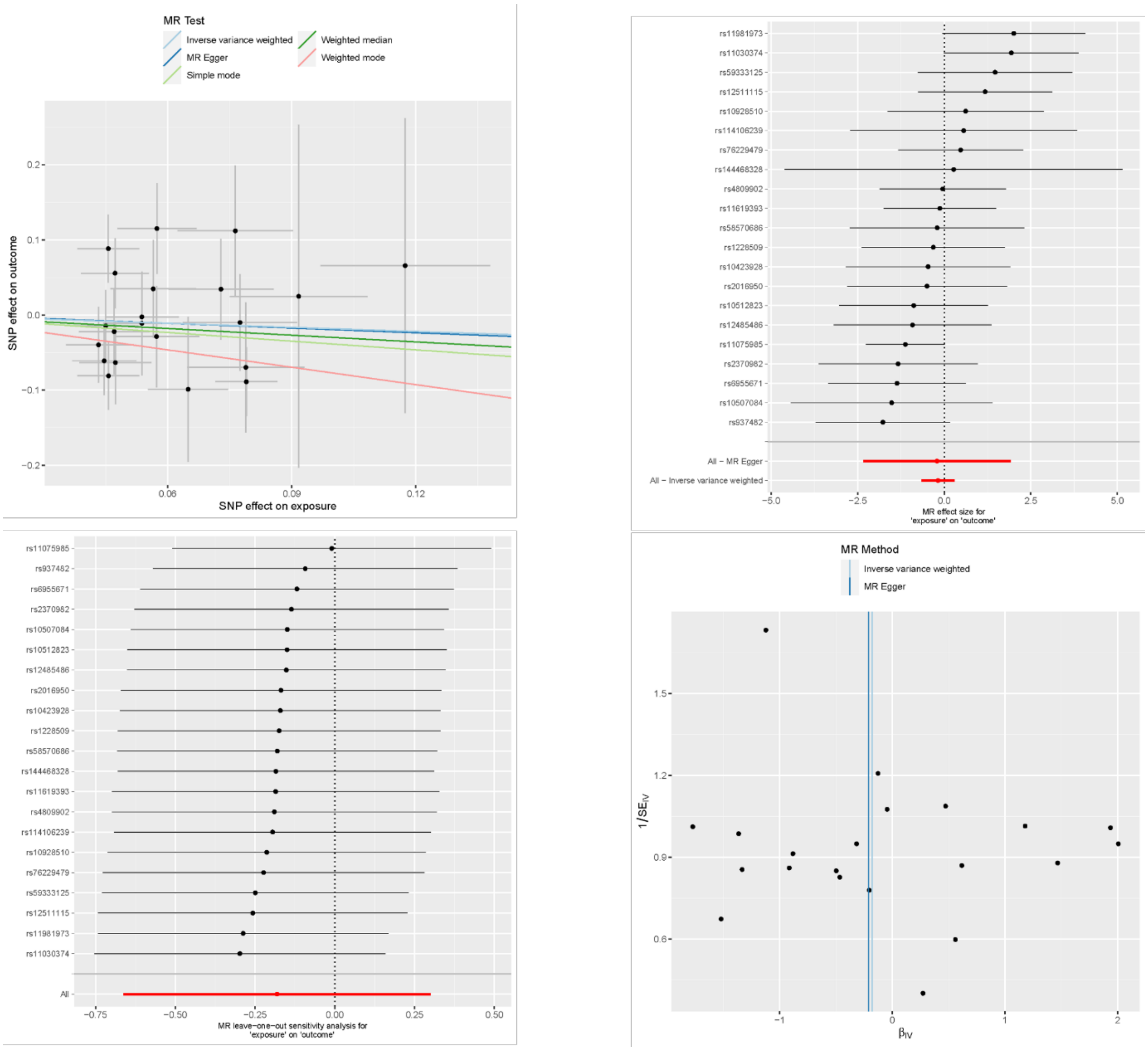

Supplement: Supplementary file 1 [file Image1.pdf]
